# Principles of Economic Rationality in Mice

**DOI:** 10.1038/s41598-017-17747-7

**Published:** 2017-12-12

**Authors:** Marion Rivalan, York Winter, Vladislav Nachev

**Affiliations:** Department of Biology, Humboldt University, Philippstr. 13, Berlin, 10099 Germany

## Abstract

Humans and non-human animals frequently violate principles of economic rationality, such as transitivity, independence of irrelevant alternatives, and regularity. The conditions that lead to these violations are not completely understood. Here we report a study on mice tested in automated home-cage setups using rewards of drinking water. Rewards differed in one of two dimensions, volume or probability. Our results suggest that mouse choice conforms to the principles of economic rationality for options that differ along a single reward dimension. A psychometric analysis of mouse choices further revealed that mice responded more strongly to differences in probability than to differences in volume, despite equivalence in return rates. This study also demonstrates the synergistic effect between the principles of economic rationality and psychophysics in making quantitative predictions about choices of healthy laboratory mice. This opens up new possibilities for the analyses of multi-dimensional choice and the use of mice with cognitive impairments that may violate economic rationality.

## Introduction

Making profitable decisions is crucial for quality of life and survival^1^. Making a decision can be defined as the selection of one option from among a set of alternatives after the integration of environmental cues, internal needs and expectations regarding the outcomes of each option^2,3^. It is an iterative process that recruits perceptive, motoric, affective and cognitive functional systems. The use of these systems can also bring vulnerabilities^4^. At a conceptual level, impairments at each step of the decision-making process can lead to the selection of options that may be disadvantageous in the long term. Although poor decisions can be categorized into different sorts (i.e., no exploration in depression, endless hand washing in obsessive compulsive disorder, substance abuse in drug addiction), due to impairments at different perceptive and/or cognitive levels, poor decision making is a key trait, co-occurring in most psychopathologies^2,5^. In the field of neuroscience, the study of poor decision making is crucial for understanding the processes leading to complex brain disorders^6^.

Perceptual decision making, which is the ability to choose an option based on the perception and analysis of sensory cues^7^, is a particularly interesting field of study examining the evolutionary roots of decision making in animals^8,9^. Sensory cues are often complex and sometimes ambiguous, and their interpretation is critical when making adaptive decisions.

Adaptive behaviour, or behaviour that maximizes a decision maker’s reproductive fitness, has been described as exhibiting biological rationality (B-rationality^10^). Similarly, when an animal makes choices that maximize expected utility, this is described as exhibiting economic rationality (E-rationality^10^). Economic rationality is mathematically defined in terms of principles such as transitivity, independence of irrelevant alternatives (IIA), and regularity, which guarantee an internal consistency of choice^10–12^. The principle of transitivity states that if ‘a’ is preferred over ‘b,’ and ‘b’ is preferred over ‘c’, then ‘a’ should be preferred over ‘c’^11^. In behavioural tests, preference is measured over many choices. As animals rarely select one choice option exclusively, this preference is partial, not absolute. Nevertheless, the principle of IIA states that the relative preference for any option in an existing choice set should not change when the choice set is increased by the addition of a new option (coined the ‘constant ratio rule’^13^, or alternatively, the ‘strong independence of irrelevant alternatives’^11^). Finally, the principle of regularity requires that the addition of a new option to a choice set should not increase the choice probability (absolute preference) for any option from the original set^11,14^. For example, if the absolute preference for A over B in binary context AB is p(A)_AB_ = 0.6, and in a trinary context ABC it is p(A)_ABC_ = 0.7 [where p(B)_ABC_ = 0.2 and p(C)_ABC_ = 0.1], this constitutes both a violation of regularity [p(A)_ABC_ > p(A)_AB_], and of IIA [p(A)_AB_/(p(A)_AB + _p(B)_AB_) ≠ p(A)_ABC_/(p(A)_ABC_ + p(B)_ABC_)].

Intriguingly, a number of studies on humans (reviewed in ref.^11^), cats^15^, birds^16–24^, insects^24,25^ and even unicellular organisms^26^ demonstrate that each of these principles are regularly violated. Studies also exist in which the principles of economic rationality were upheld, e.g., in monkeys^27^, ants^28^, and birds^29,30^.

One argument forwarded to explain apparent violations of rationality is that state-dependence effects must be considered, e.g., when an animal prefers a small immediate reward option when its current energy reserves are low and a large delayed option when its reserves are high^21^. State dependence can lead to violations of economic rationality, especially when the value of an option depends on the context of all available options, including those not chosen^31–33^. Theoreticians have even proposed the removal of the principles of regularity and transitivity as necessary conditions for rational choice^32,33^, which amounts to arguing that only violations of B-rationality (fitness maximization) and not E-rationality (utility maximization) should be labelled as irrational behaviours. However, with one exception^23^, violations of the principles of economic rationality have only been demonstrated when options vary in more than one dimension (e.g., delay and probability of reward), where interactions between dimensions can occur because of nonlinearities in the evaluation of each dimension^34,35^.

Such nonlinearities are described by Weber’s law, which supports the general observation in perception that discrimination thresholds are a constant proportion of stimulus magnitude^34^. Indeed, it is consistent with Weber’s law (or proportional processing) that, in most animal species, responses to differences in intensity between physical stimuli indicate that such intensity differences are represented proportionally rather than linearly^34^. For instance, the addition of two peanuts to a bowl holding 15 peanuts will barely be noticed, whereas two extra peanuts in a bowl holding five peanuts will be readily perceived. The proportional processing of sensory information can be demonstrated by fitting a psychometric function into the choice probabilities experimentally determined. A psychometric function then describes how the probability to choose the better of two options increases as the difference between two value stimuli increases. This function usually has a sigmoid shape^36,37^. Importantly, knowing how the probability of choice changes as the difference between two stimuli increases allows for quantitative rather than simply qualitative predictions about expected performance in future choice experiments.

Rodents are notably absent from the list of animals in which the principles of economic rationality have been tested. Rats and mice have long been used in the study of the diverse aspects of motor, sensory, affective and cognitive functions such as learning, memory and the processing of rewards and punishments^38–40^. This includes studies of the neural mechanisms underlying perceptual functions in cognitive tasks^41,42^, complex decision-making abilities^43,44^ and decision making based on (multi)sensory input^45–47^. Thus, rodents hold promise for investigating how decision-making circuits function in healthy conditions and when disrupted by disease^48^. In order to increase the ecological validity of laboratory studies, automated home cage setups have been introduced^49–52^, in which operant testing modules are placed inside the home cages of group-living mice or rats. With such setups, it is possible to screen cognitive and affective abilities in an environment in which rodents are undisturbed and follow their own cycle of activity and level of motivation for extended periods of time.

We investigated whether mice exhibit economic rationality by testing the principles of transitivity, IIA and regularity during choices made in an automated home cage experimental system. Mice were given access to dispensers delivering rewards (drinking water) that differed in one of two dimensions, namely volume or probability. We evaluated stochastic transitivity within sets of binary choice conditions for each reward dimension, volume or probability^29^. Mice could choose between four dispensers, two of which (binary) presented a medium or high-quality reward, whereas the other two presented only (irrelevant) small or zero rewards. While mice kept visiting all dispensers even after long exposure to constant conditions, they still exhibited underlying preferences that could be statistically detected.

We evaluated the principle of IIA by comparing preferences in binary and trinary choice conditions. Here, the difference between the binary and the trinary choice conditions was an additional irrelevant option that was objectively inferior in value to the other two options^29^. Over the course of all experiments, mice were required to choose between many pairs of volumes or probabilities, with differences within these pairs ranging from zero to small to large. We used the recorded relative preferences of the mice in each choice condition to fit psychometric functions for the discriminative ability of volume and probability. These functions then allowed us to estimate the probability that a mouse will choose the larger or better of the two options as a function of the difference in magnitude between these two options. Finally, since our experimental setup allowed us to test animals’ decisions in a social environment, we performed control tests that ascertained that the decisions were not influenced by social learning. This was accomplished by reassigning dispensers to mice, so that twice as many animals were using same dispensers as used previously, and then evaluating whether this altered choice behaviour.

## Animals, Materials and Methods

### Subjects

The main experiments were carried out with female C57BL/6NCrl mice (Charles River, Germany, *N*_*total*_ = 19). A pilot study was conducted using eight male mice^53^. Upon delivery, mice were five weeks old. They were then housed together and marked with unique Radio Frequency Identification tags (RFID: 12 × 2.1 mm, 125 kHz, Sokymat, Germany) at six weeks of age. At seven weeks of age, mice were transferred to the automated group home cage for the main experiment. Pellet food was always accessible from a trough in the cage lid, and water upon nose pokes (and individual schedule) from the operant modules of the automated group home cage. Light conditions during the experiments were 12:12 LD and climatic conditions were 23 ± 2 °C and 50–70% humidity.

### Ethics statement

The experimental procedures described here were designed to allow for maximal animal welfare. Animals lived undisturbed as a group within their home cage. Briefly, data collection was performed using automated detection while animals voluntarily visited the water dispensers to drink. The health of the animals was monitored daily. Due to the observational nature of the study, the experimental procedure did not cause any damage, pain, or suffering to the animals. The animals were not sacrificed at the conclusion of the study. This study was performed under the supervision of the animal welfare officer (*Tierschutzbeauftragter*) heading the animal welfare committee at Humboldt University that approved the procedures. Experiments followed national regulations in accordance with the European Communities Council Directive 10/63/EU.

### Cage and dispenser system

**Figure 1:**
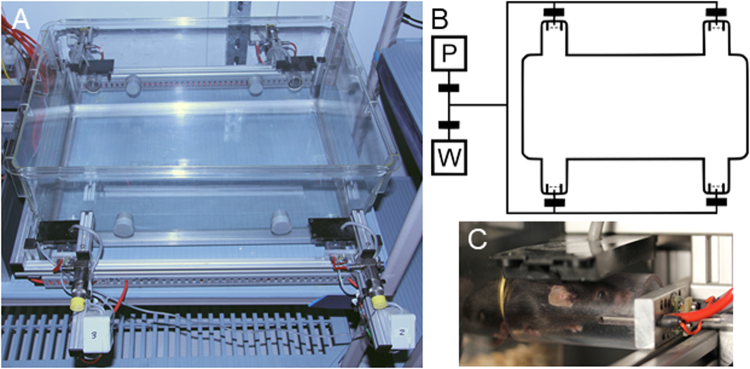
Automated group home cage. (**A**) Photo of an automated group home cage. The four computer-controlled water dispensers (number 2 and 3 are visible in the front, number 1 and 4 are in the back) sit outside the home cage and are accessible through holes drilled in the cage’s wall (four other holes are closed by rubber plugs). Above each dispenser is a RFID-sensor (rectangular black box). (**B**) Schematic representation of the tubing system (top view). The dispensers in the four corners of the cage (rounded rectangle) are connected via tubes (continuous lines) to a stepping motor syringe pump (P) and water reservoir (W). Water flow is controlled with the pump and pinch valves (large black rectangles). Rewards are triggered when a transponder-identified mouse makes a nose poke between an infrared emitter and receiver (small rectangles with dashed lines, indicating IR beams). (**C**) A close-up of a mouse visiting a dispenser.

Mice were housed together in an automated group home cage (612 × 435 × 216 mm, P2000, Tecniplast, Italy) with *ad libitum* access to pellet food (V1535 chow pellet, maintenance food, ssniff, Germany), woodchip bedding (AB 6, AsBe-wood, Germany), and enriched with two grey PVC tubes and paper towels as nesting material. The cage was outfitted with four computer-controlled liquid dispensers (Fig. 1). Dispenser visits were detected by infrared beam-break sensors, and the identity of the mouse was detected via RFID-sensor at each of the dispensers. Reward delivery was controlled by a syringe pump using a gas-tight Hamilton glass syringe (Series 1025). Dispenser spouts were connected to the pump through a system of pinch valves and tubes (Fig. 1). This arrangement made it possible for water delivery at a specific dispenser to be restricted to certain individuals, and for the amount of a water reward to be under experimental control at all times. Rewards consisted of droplets of water from the dispenser spout that mice removed by licking the tip of the spout. Cage bedding was changed and animals weighed on a weekly basis, always during the light phase and at least an hour before commencement of the testing session. Data were recorded automatically and stored on a computer, using custom-written software in C#, based on the.NET framework. Time-stamped nose poke events were recorded for each individual, with their corresponding dispensers and amount of water reward delivered.

### Experimental schedule

In all experimental phases, the drinking session commenced at the onset of the dark phase and ran for 18 h, concluding six h after the end of the dark phase. Nose pokes outside of the drinking session were not rewarded. The dispensers delivered rewards with varying volumes and probabilities on an individual basis dependent on the experimental phase. Although mice shared the same cage and dispensers, different individuals were not necessarily in the same experimental phase. The aim was to perform the main experiments with eight mice simultaneously within one cage. Experiments were conducted consecutively with two groups of mice (Supplementary Figure S1). These groups were: the ‘volume first’ group (exploratory phase: *N* = 10, main experiment: *N* = 8 successfully pre-trained mice) and the ‘probability first’ group (exploratory phase: *N* = 9, main experiment: *N* = 7 successfully pre-trained mice). Exclusion criteria for the animals are described below. In all phases of the experiment, if a mouse obtained less than 1,000 µL of water during a drinking session, two water bottles were placed in the automated home cage for 10–20 min during the light phase, mice were awakened and allowed to drink until they voluntarily stopped. Usually only the individuals that failed to meet this criterion approached and drank from the bottles.

#### Exploratory phase

Animals were transferred to their new home cage during the light phase, 1–2 hours before the initial drinking session of the exploratory phase. During the exploratory phase, all dispensers gave constant rewards of 20 µL per nose poke. If a mouse consumed more than 700 µL of water at the end of the first drinking session of the exploratory phase, it proceeded to the training phase; otherwise, it repeated this phase during the next drinking session. The exploratory phase was required to ensure that mice learned where and how to obtain water within the cage.

#### Training phase

In the ‘volume first’ group, the rewards began during the initial training phase as 33 µL of water, but were then reduced to 10 µL, in order to increase the number of choices each mouse was required to make. The volume in the training phase of the ‘probability first’ group was constant at 10 µL. The probability remained at 30% during all training phases. Lower probabilities ensured that mice made more visits and remained motivated. The training phases continued for two to five days until all mice fulfilled the criterion of consuming more than 1000 µL of water in one drinking session. The training phase was required to habituate the mice to the background dimensions (volume or probability) that would be used in the following discrimination experiments.

#### Discrimination experiments

Two types of discrimination experiments were performed: volume discrimination and probability discrimination. The ‘volume first’ group was initially tested for volume discrimination and then for probability discrimination, whereas the ‘probability first’ group had the reverse order. The general conditions were the same for both types of discrimination experiments: for each mouse, one of the dispensers had high profitability, another had medium profitability, and the remaining two dispensers had an identical low profitability. There were five possible reward types. These were, in order of decreasing profitability, A, B, C, D, and 0; with A = 22 µL, B = 19 µL, C = 14 µL, D = 6 µL, 0 = 0 µL in the volume discrimination experiments, and A = 80%, B = 70%, C = 50%, D = 20%, 0 = 0% in the probability discrimination experiments. All possible combinations of low, medium and high profitability were tested, resulting in six ‘binary choice’ conditions: AB0, AC0, AD0, BC0, BD0, and CD0 (0 designating the non-rewarding dispensers), and four ‘trinary choice’ conditions: ABC, ABD, ACD, and BCD. The three-symbol code gives the reward types in decreasing order of profitability. For example, for the ACD condition in the volume discrimination experiment, one dispenser delivered 22 µL (high profitability), another dispenser delivered 14 µL (medium profitability), and the remaining two delivered 6 µL (low profitability). Comparing the relative preference for the option with high profitability in the binary and trinary conditions was done as a test of regularity.

The reward probabilities were set to 30% for all dispensers in the volume discrimination experiments, in order to make return rates comparable for both dimensions. The reward volumes were set to 10 µL in the probability discrimination experiments. In the volume experiment involving the ‘volume first’ group, whether a reward was given to a mouse (upon nose poke) was decided by drawing samples with 30% probability. In all subsequent experiments, rewards were drawn from fixed pseudo-random repeating sequences. These sequences were: 11101111101101111110 for 80%, 11011101110101101110 for 70%, 10110101101001001010 for 50%, 10010100100001001000 for 30%, and 10001000010001000000 for 20%, where 1 is a rewarded nose poke and 0 is an unrewarded nose poke.

**Figure 2:**
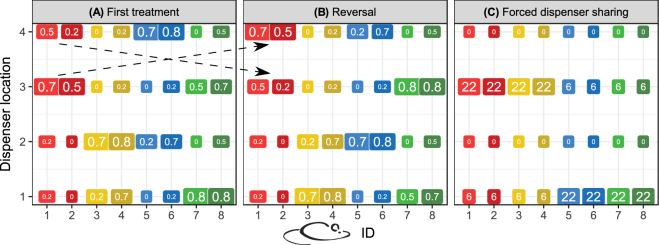
An example of dispenser distribution among mice. (**A**) In each condition of the main experiment, mice (color-coded 1 through 8) were given a reward profitability (indicated by rounded rectangle size) that encouraged their even distribution among dispensers. For example, dispenser 1 had high profitability for mice 7 and 8 (pair 4, large green rectangles), medium profitability for mice 3 and 4 (pair 2, intermediate yellow rectangles), and low profitability for the remaining mice (pairs 1 and 3, small red and blue rectangles). (**B**) Each condition was followed by a reversal session during the next night, in which the high and medium-profitability dispensers were spatially inverted (dashed arrows), while the low-profitability dispensers remained the same. (**C**) During the forced dispenser sharing condition (social learning test), all mice shared the same low-profitability dispensers. The mice were split in two subgroups (mice 1–4 and mice 5–8), so that mice within each subgroup but not between subgroups shared the same high and medium-profitability dispensers, 1 and 3. This condition was maintained for two days, with a reversal on the second day (not shown). Numbers inside rectangles give either reward probabilities (**A**,**B**) or volume in microliters (**C**).

Mice were paired so that the mice in each pair shared the same high and medium profitability dispensers each night. The pair shared these two dispensers with another pair of mice, for which the medium and high profitability dispensers were spatially inverted. Thus, each night each dispenser had a high profitability for the mice in one pair, a medium profitability for the mice in another pair, and a low profitability for the mice in the remaining two pairs (Fig. 2). The pairs of mice remained the same throughout the experiment, until the social learning tests (see next section). One mouse was unpaired in the ‘probability first’ group.

The sequence of conditions (binary and trinary) was randomized for each individual. Thus, although the mice in a pair shared dispensers, they did not usually experience the same type of reward from this dispenser. As a control for positional biases, each given condition was followed by a reversal on the following day, so that the high and medium-profitability dispensers were spatially inverted for each mouse, whereas the two low-profitability dispensers remained unchanged. After reversal, the experiment continued with the next condition, with random distribution of the dispensers among the pairs of mice following the previously described constraints (Fig. 2). Over each 20-session discrimination experiment [(6 binary conditions + 4 trinary conditions) × 2 because of the reversal conditions], each mouse experienced each dispenser as a low-profitability dispenser between eight and 12 times. If an electrical or mechanical malfunction occurred, the data from the failed condition and its reversal were discarded and mice proceeded with the next condition, until all conditions (including those that had originally failed) were completed. A failure occurred twice in each of the two groups of mice. After the first discrimination experiment, another four-day training phase (rewards with 10 µL volume and 30% probability) was given, followed by the second discrimination experiment. Finally, several additional tests of social learning were performed (see below) and the mice were returned to the animal facility.

#### Social learning tests

By using group housing, we provided a more natural environment for our mice, while simultaneously increasing the efficiency of experimentation. As each mouse was designated an individual sequence of experimental conditions, the effect of the condition sequence was controlled within the main experiment. However, as there were eight mice and four dispensers, it was necessary to pair mice to share their high and medium-profitability dispensers with three other mice (Fig. 2A,B). In order to assess potential social influences on discrimination, we increased the possibilities for social learning during additional volume discrimination sessions. These sessions aimed to test whether extensive training had increased performance in volume discrimination to levels comparable to those seen in the probability discrimination. In the ‘volume first’ group, after the end of the probability discrimination experiment, six binary volume discrimination conditions with corresponding reversals were repeated, including a final AD0 condition that was given for two sessions (the second session was a reversal). In this forced dispenser sharing condition, all mice had the same two low-profitability dispensers (spatial positions 1 and 3), and for half of these mice dispenser 1 had a high profitability and dispenser 3 had a medium profitability, whereas for the other half the dispenser profitabilities were spatially inverted (Fig. 2C). Thus, in contrast to the main experiment, mice could potentially profit to an increased extent if they rely on the preferences of other mice when making choices. There was simultaneously higher competition for access to the water dispensers. In summary, the AD0 condition was experienced by the mice three times: 1) In the original volume discrimination experiment, 2) in the repeat experiment, and 3) in the forced dispenser-sharing condition. This was done to control for a potential increase in discrimination performance due to experience. Comparison of 1) and 2) shows whether time affected choice. Comparison of 2) and 3) shows whether sharing the active dispensers with all mice and the most profitable dispenser with half of the mice affected choice.

In the ‘probability first’ group, mice also experienced these three AD0 conditions, although in a different order. Immediately after the end of the second discrimination experiment, mice were given a forced dispenser-sharing AD0 condition (test and reversal). The mice were subsequently given one night of ad libitum access to water before they completed a final AD0 condition (test and reversal). In this group, the mice only repeated the AD0 condition and no other volume discrimination conditions.

### Data Analysis

In each drinking session (18 h in duration, of which 12 h during the dark phase), mice made an average of 585 ± 128 (mean ± SD, *N* = 20 days per group, *N* = 15 mice) nose pokes at the different dispensers. Based on experience with this system and on preliminary data evaluation, we excluded the first 250 choices (nose pokes) from analysis, in order to focus on post-acquisition performance (Supplementary Figs S1–S3). For each mouse and each condition, we calculated the *relative intensity*, *discrimination performance* and *sampling rate*. The relative intensity was calculated as the absolute difference between the volumes (or probabilities) of the high and medium-profitability dispensers, divided by the mean volume (or probability) of the high and medium-profitability dispensers^54^. The relative intensity can be understood as the reward intensity by which two options differ. The discrimination performance was calculated over the two presentations of the same condition (original and reversal) as the total number of nose pokes at the high-profitability dispenser divided by the total number of nose pokes at the high and medium-profitability dispensers (nose pokes at the low-profitability dispensers were ignored). The sampling rate was calculated over the two presentations of the same condition as the number of nose pokes at the low-profitability dispensers divided by the total number of nose pokes at all dispensers. As seen in previous studies^54–56^, we expected the preference for the high-profitability dispenser (discrimination performance) to increase as its return increasingly differed over that of the medium-profitability dispenser (relative intensity). In contrast, we expected the nose pokes in the irrelevant (low-profitability) dispensers (considered as a measure of sampling rate) to be independent from the relative intensity between medium and high-profitability dispensers. Statistical tests were performed in R 3.1.1^57^.

#### Tests for economic rationality

Testing for stochastic transitivity entailed comparisons within each transitivity set of three binary conditions^29^ with different relative intensities, e.g., set {ABC}, consisting of AB0 (relative intensity = 0.13), BC0 (0.33), and AC0 (0.46). In general, discrimination performance increases (nonlinearly) with relative intensity^54^. Thus, in each transitivity set (Table 1), the discrimination performances in the conditions with the highest and intermediate relative intensities were compared to test strong stochastic transitivity (e.g., whether discrimination performance in AC0 was lower than in BC0), and the discrimination performances of the conditions with the intermediate and lowest relative intensities were compared to test moderate stochastic transitivity (e.g., whether performance in BC0 was lower than in AB0^29^). In both cases, non-transitivity is indicated by discrimination performance significantly decreasing with relative intensity. A paired one-tailed *t*-test was used for these comparisons, and a single sample one-tailed *t*-test was used to determine whether the discrimination performance in the condition with the highest relative intensity significantly differed from 0.5, which is required to test for weak stochastic transitivity. The same qualitative results were obtained using non-parametric tests and when controlling for the false discovery rate where appropriate^53^.

**Table 1:** Tests for stochastic transitivity in volume and probability discrimination experiments in female mice (*N* = 15).

Reward dimension	Test for stochastic transitivity	Transitivity set	Condition with highest relative intensity	Alternative condition	*t* ^a^	*p* ^b^
**Probability**	Weak	{ABC}	AC0	—	4.99	**<0.001**
**Probability**	Weak	{ACD}, {ABD}	AD0	—	10.20	**<0.001**
**Probability**	Weak	{BCD}	BD0	—	8.83	**<0.001**
**Volume**	Weak	{ABC}	AC0	—	2.85	**0.006**
**Volume**	Weak	{ACD}, {ABD}	AD0	—	5.05	**<0.001**
**Volume**	Weak	{BCD}	BD0	—	4.74	**<0.001**
**Probability**	Moderate	{ABC}	AC0	AB0	4.31	**<0.001**
**Probability**	Moderate	{ABD}	AD0	AB0	9.02	**<0.001**
**Probability**	Moderate	{ACD}	AD0	AC0	4.68	**<0.001**
**Probability**	Moderate	{BCD}	BD0	BC0	4.90	**<0.001**
**Volume**	Moderate	{ABC}	AC0	AB0	0.98	0.171
**Volume**	Moderate	{ABD}	AD0	AB0	3.94	**0.001**
**Volume**	Moderate	{ACD}	AD0	AC0	3.40	**0.002**
**Volume**	Moderate	{BCD}	BD0	BC0	2.62	**0.010**
**Probability**	Strong	{ABC}	AC0	BC0	1.82	**0.045**
**Probability**	Strong	{ABD}	AD0	BD0	1.17	0.131
**Probability**	Strong	{ACD}	AD0	CD0	1.97	**0.035**
**Probability**	Strong	{BCD}	BD0	CD0	1.37	0.096
**Volume**	Strong	{ABC}	AC0	BC0	0.59	0.284
**Volume**	Strong	{ABD}	AD0	BD0	1.37	0.096
**Volume**	Strong	{ACD}	AD0	CD0	0.11	0.457
**Volume**	Strong	{BCD}	BD0	CD0	−1.29	0.110^c^

^a^ *t*-test statistics are from single sample one-tailed *t-*tests against 0.5 for weak transitivity and from paired one-tailed *t*-tests with Welch’s correction for unequal variance for moderate and strong transitivity.

^b^Values smaller than 0.05 are given in bold. The same qualitative results were obtained with non-parametric tests^53^. Since a violation of moderate and strong stochastic transitivity would entail a significantly negative *t* value and as only a single observed value was negative, uncorrected *p*-values are reported.

^c^The *p*-value reported here is for the hypothesis BD0 < CD0. All other *p*-values are for hypotheses of the form: condition with highest relative intensity > alternative condition.

In order to examine a potential effect of the additional choice option in trinary conditions (independence of irrelevant alternatives), a paired two-tailed *t*-test was used to compare discrimination performance in corresponding binary and trinary conditions, involving the same high-profitability and medium-profitability options (e.g., ABC and AB0). Finally, as a test of the principle of regularity, a paired one-tailed t-test was used to ascertain whether the discrimination performance in binary conditions was lower than the number of visits to the high-profitability dispenser divided by the total number of visits in trinary conditions^53^.

#### Psychometric analysis (fitting performances for each of the reward dimensions)

In order to compare the overall performance in the two groups of mice for each of the reward dimensions (volume and probability) we fitted individual psychometric curves that illustrated how the probability to choose the better option (discrimination performance) increased with relative intensity. The experimental values for the two reward dimensions were specifically chosen to have relative intensities that were as similar as possible, which equates to similar return rates for both dimensions. For a good fit of the psychometric function, a wide range of tested intensities is required^58^. Of special interest are points of high intensity, for which we examined the sampling rates in each of the six binary conditions. One minus the sampling rate was calculated in each condition as an estimate of maximal discrimination performance. The non-linear least-squares nls function in R was then used to fit individual psychometric functions using the observed discrimination performances at the different relative intensity levels^53,54^. The lapse rate estimates were constrained between twice the sampling rate estimate and 1, the thresholds between 0 and 2, and the slopes between 0 and 3 (in order to prevent unrealistically-high estimates, as typical slope values are around 1). The starting values were the minimum constraint for the lapse rate, 0.5 for the threshold, and 0.5 for the slope. Once the individual estimates for the threshold and slope were obtained, generalized linear mixed models were used (MCMCglmm package in R^59^) to test for effects of reward dimension (volume or probability) and experimental group (volume first or probability first), with mouse as random effect. In this and all other models, parameter-expanded priors were used^53^. Estimates of the fixed effects (*β*) are given as averages of the posterior with 95% credibility intervals, based on 1,000 simulations^59^. The Markov chains were iterated 1,300,000 times, with a burn-in period of 300,000 and a thinning interval of 1,000. Significance is reported as posterior probability (*pMCMC*), based on the overlap of the posterior distribution with 0. Since slope estimates were censored at 3.00, the cengaussian family was used for the slope model and the Gaussian family for the threshold model. Finally, rather than testing lapse rate, the focus was on its close relative, the sampling rate. Lapse rate is a single estimated value per animal and the sampling rate was measured once per each condition, which allows other effects to be tested, such as the profitability of the irrelevant option, etc.

#### Sampling rate (frequency of nose pokes at the irrelevant options)

Generalized linear mixed models (MCMCglmm package in R) were used to test whether sampling rate was affected by experimental group (volume first or probability first), reward dimension (volume or probability), relative intensity, and profitability of the irrelevant options (all four variables entered as fixed effects), with mouse as random effect. The profitability of the irrelevant options was entered in the model as proportion of the overall highest profitability, i.e., 0 in the binary conditions, 0.25 in conditions ABD, ACD, and BCD, and 0.625 in condition ABC.

#### Social learning tests

A paired two-tailed *t*-test was used to compare discrimination performances and sampling rates in the three different AD0 conditions: Original volume discrimination experiment, in the repeat experiment and in the forced dispenser-sharing condition. A difference between the original and repeat conditions would indicate a general learning effect, and a difference between the control and forced dispenser-sharing conditions would indicate an effect of social learning or interference competition. The datasets generated during and/or analysed during the current study are available in the Zenodo repository^53^: https://doi.org/10.5281/zenodo.1014052.

## Results

### Tests for economic rationality: Transitivity and independence of irrelevant alternatives

**Figure 3:**
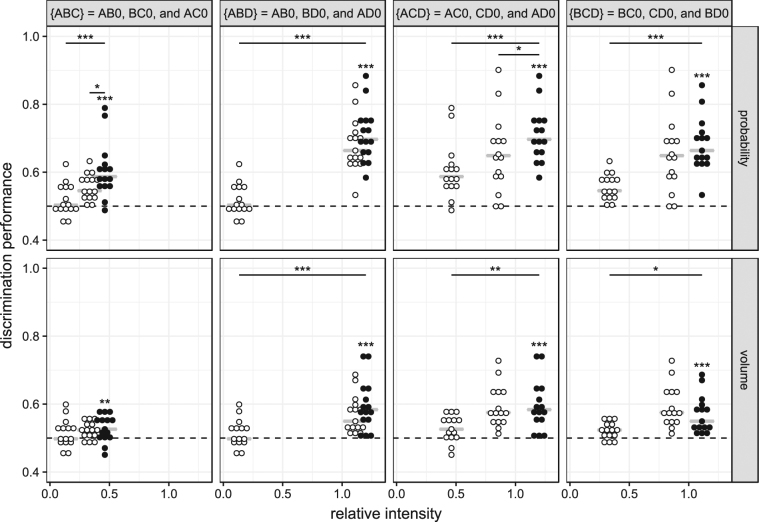
Discrimination performance in different transitivity sets. Circles show the individual discrimination performances of all mice (*N* = 15) and grey lines the medians. Columns correspond to the different transitivity sets and rows, namely to the reward dimensions (probability or volume). In each transitivity set, the average discrimination performance in the binary condition with the highest relative intensity (closed circles) was significantly higher than chance level (dashed line; single sample one-tailed *t*-test). Comparisons of the lowest and the highest conditions and of the intermediate and highest conditions are shown with black horizontal bars (paired one-tailed Welch’s *t*-test; see Table 1 for complete statistics). Volume discrimination experiments: A = 22 µL, B = 19 µL, C = 14 µL, D = 6 µL, 0 = 0 µL. Probability discrimination experiments: A = 80%, B = 70%, C = 50%, D = 20%, 0 = 0%. Probability was held constant at 30% in the volume discrimination experiments and volume was held constant at 10 µL in the probability discrimination experiments. ^*^ *p < *0.05, ^**^ *p < *0.01, ^***^ *p < *0.001.

No violation of transitivity was found in either the volume or probability discrimination experiments (Fig. 3). The discrimination performances of all conditions with the highest relative intensity were significantly different from the chance level of 0.5 (Table 1, Fig. 3). This satisfied the requirement for weak stochastic transitivity in all transitivity sets. Furthermore, in all transitivity sets, the requirement for moderate transitivity was also satisfied. The conditions with intermediate relative intensities had higher discrimination performances than the conditions with the lowest relative intensities (the difference was significant in seven of eight comparisons, Table 2). When comparing discrimination performances between the conditions with the highest and intermediate relative intensities, the difference was seen in the direction consistent with transitivity in seven of eight comparisons, with two of the seven differences being significant (Table 2). The only observation in the opposite direction was in set {BCD} in the volume discrimination experiment; however, this difference was not significant (Table 1). Thus, the requirement for strong stochastic transitivity was also satisfied, as discrimination performance did not significantly decrease with relative intensity.

**Table 2:** Comparison of discrimination performance in binary and trinary conditions.

Reward dimension	Binary condition	Trinary condition	*t* ^a^	*p*
Probability	AB0	ABC	0.72	0.486
Probability	AB0	ABD	0.92	0.371
Probability	AC0	ACD	−1.16	0.265
Probability	BC0	BCD	−1.85	0.085
Volume	AB0	ABC	−0.60	0.557
Volume	AB0	ABD	0.23	0.826
Volume	AC0	ACD	0.59	0.562
Volume	BC0	BCD	0.72	0.484

^a^ *t*-test statistics from two-tailed paired Welch’s *t*-tests. The same qualitative results were obtained with non-parametric tests^53^.

**Figure 4:**
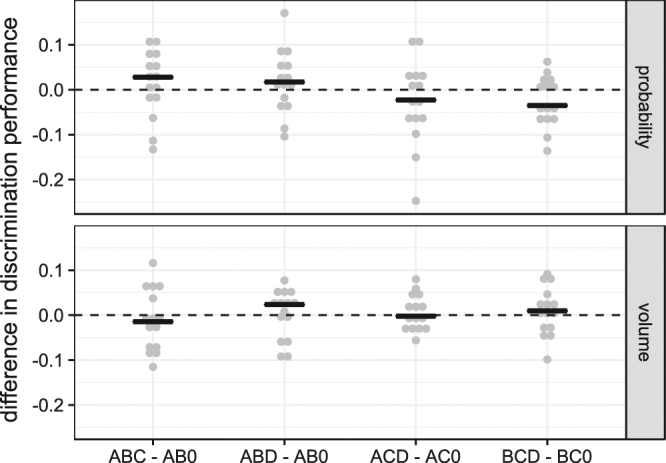
Difference in discrimination performance between binary and trinary conditions. Circles show the individual differences in discrimination performance (for the given conditions) of each individual mouse (*N* = 15) and the lines show the medians for each set. Rows correspond to reward dimension (probability or volume). The dotted line at 0 indicates a lack of difference in discrimination performance between the two conditions.

In both volume and probability discrimination experiments, the discrimination performance did not change significantly between binary and trinary conditions in any condition. This indicates compliance with the principle of IIA (Table 2, Fig. 4), and regularity (Supplementary Fig. S4).

### Psychometric analysis (fitting performances for each of the reward dimensions)

**Figure 5:**
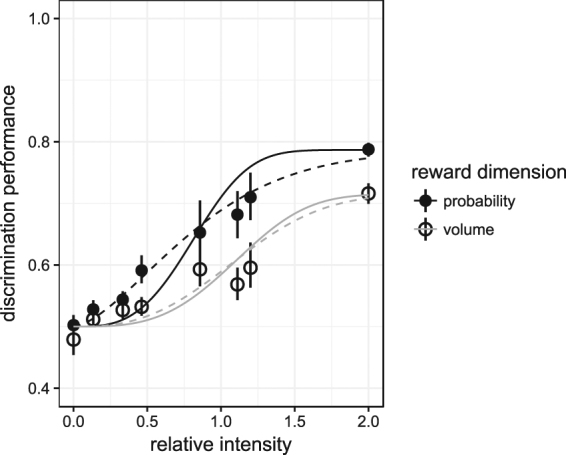
Psychometric functions for probability (black lines, closed symbols) and volume (grey lines, open symbols) discrimination. Symbols show average discrimination performance for *N* = 15 mice. Continuous lines represent the psychometric functions with parameters computed by averaging the parameters of individual psychometric functions (Methods). For the dashed lines, the psychometric function was computed from the pooled data from all mice. This underestimates the slope of the psychometric function of a typical mouse (slopes of the dashed curves are 0.72 and 0.85 for probability and volume, respectively), but yields a curve that predicts discrimination performance of an average group of mice^36^. The whiskers show the mean standard errors from bootstraps.

On average, the threshold (inflection point) in the probability experiment was at a significantly lower intensity than in the volume experiment; however, no significant difference was observed in the slope of the psychometric functions (Fig. 5, Table 3; Supplementary Fig. S6; Supplementary Table S7). Furthermore, there was no effect of experimental sequence (volume first or probability first, Table 3). Thus, mice showed a significantly better discrimination performance for probability than they did for volume.

**Table 3:** Summary of fixed effects from generalized linear mixed-effects models (GLMMs) of threshold and slope estimates from Supplementary Table S7.

Dependent variable	Fixed effects	*β*	l-95%	u-95%	*pMCMC*
Threshold	Intercept	0.849	0.689	1.018	<0.001
	Reward dimension (Volume)	0.283	0.110	0.435	0.002
	Group (Volume first)	0.059	−0.242	0.154	0.570
Slope	Intercept	2.830	2.481	3.001	<0.001
	Reward dimension (Volume)	−0.083	−0.324	0.064	0.176
	Group (Volume first)	0.103	−0.054	0.450	0.206

Shown are estimates of the fixed effects (***β***), along with the lower and upper 95% credible intervals, and the posterior probability (*pMCMC*).

### Sampling rate (frequency of nose pokes at the irrelevant options)

As predicted, the sampling rate did not increase with relative intensity (Table 4). However, it did increase as the profitability of irrelevant options increased, and was generally higher in the volume than in the probability discrimination experiment (Table 4). The sampling rates between the two groups did not differ significantly, as there was no group effect (Table 4).

**Table 4:** Summary of fixed effects from a generalized linear mixed-effects model of sampling rate.

Fixed effects	*β*	l-95%	u-95%	*pMCMC*
Intercept	−1.185	−1.360	−0.994	<0.001
Relative intensity	−0.089	−0.215	0.040	0.176
Reward dimension (Volume)	0.231	0.136	0.310	<0.001
Group(Volume first)	−0.051	−0.243	0.173	0.608
Profitability of irrelevant options	1.673	1.417	1.914	<0.001

Shown are estimates of the fixed effects (**β**), along with the lower and upper 95% credible intervals, and the posterior probability (pMCMC).

### Social learning test

Mice in both groups increased their discrimination performances from the original AD0 volume condition to the repeated AD0 volume condition at the conclusion of the main experiments (Table 5). However, compared to this improved baseline performance, no further improvement was seen when mice shared their high profitability dispenser with three mice rather than with only one mouse. In contrast, only the mice in the volume first, but not in the probability first group, significantly decreased their sampling rates from the original condition to the control condition. Finally, when mice from the probability first group shared the same rewarding dispensers with all other mice in their group, their sampling rates increased compared to the control condition, in which they shared their rewarding dispensers with only three mice (Table 5).

**Table 5:** Comparison of discrimination performances and sampling rates in the control AD0 volume condition versus the main experiment AD0 condition or the forced dispenser-sharing AD0 condition.

Parameter	Experimental group	Alternative AD0 condition^a^	*t* ^b^	*P*
Discrimination performance	Probability first (*N* = 7 mice)	Main	6.29	**0.001** ^**c**^
	Probability first	Forced dispenser sharing	0.37	0.723
	Volume first (*N* = 8 mice)	Main	11.03	**<0.001**
	Volume first	Forced dispenser sharing	−0.28	0.787
Sampling rate	Probability first	Main	1.43	0.203
	Probability first	Forced dispenser sharing	4.70	**0.003**
	Volume first	Main	−3.87	**0.006**
	Volume first	Forced dispenser sharing	−0.31	0.766

See Fig. 2 and Methods for explanation of conditions.

^a^The comparisons are always against parameters from the control condition.

^b^ *t*-test statistics are from paired two-tailed Welch’s *t*-tests.

^c^Values smaller than 0.05 are given in bold. The same qualitative results were obtained with non-parametric tests and with corrections for false discovery rate^53^.

## Discussion

The choice behaviour of the mice in this study was consistent with the principles of economic rationality. This demonstrates the usefulness of normative models of choice and in particular the effectiveness of principles for generating qualitative predictions about choice preferences in natural settings. The experimental design deviated from the classical tests of transitivity, regularity, and independence of irrelevant alternatives (IIA^10–12^), as in our experiments there were in effect four food options rather than two or three. For transitivity and IIA tests, the visits at the non-rewarding dispensers could simply be ignored in the calculation of discrimination performance, demonstrating that tests of these effects can be extended to multiple options. The only test of economic rationality that took visits at the irrelevant options into account was the test of regularity. However, the expectation for regularity remained that the choice rate to any option relative to all options available would not increase with the addition of new options (whether one or two new options were added) to the choice set. Indeed, the choices of each single mouse were consistent with regularity (Supplementary Fig. S4), showing that this test could also be extended to include more than three options. Based on our results, we argue, as other authors have before us^29^, that when the physiological and informational state of the decision makers is properly controlled, the compliance with the principles of economic rationality is the norm, rather than the exception.

Since the effect of state-dependence on violations of economic rationality has been thoroughly discussed elsewhere^21,31–33^, we next examined the psychophysical effects. The results indicate that, as seen with perceptual quantities such as volume and sugar concentration^34,54^, reward probability also appears to be processed proportionally, rather than linearly; therefore, discrimination performance and choice can be described and predicted using a psychometric curve (Fig. 5). Furthermore, although probability is a reward dimension that can only be indirectly estimated over a number of choices, mice initially showed a better discrimination performance for differences in probability than for differences in volume (Fig. 5; Supplementary Table S5). This is surprising, because if the currency being optimized were the average amount of water per visit, then no difference should have been seen between the psychometric curves for volume and probability. One might argue that in a natural habitat with stochastic variation of resource availabilities it is important to capture these probabilities. However, after several weeks mice were able to improve their discrimination performance for volume beyond that of the performance for probability as they became more attuned to the volume dimension (Table 5). Whether performance in probability discrimination also benefits from extensive training was not tested here. Thus, it appears that at least naïve laboratory mice tend to respond to differences in probability more strongly than to differences in volume. This may be because under natural conditions the volume of water droplets, e.g., in dew, is likely to be a less-predictive reward dimension than the availability of water.

Psychometric functions make quantitative predictions about discrimination performance for any two options differing in volume or probability for mice under similar conditions and levels of training. For example, prior to this study, a pilot experiment was performed with eight male C57BL/6NCrl mice, using a similar protocol as that here used in the ‘volume first’ group, but only testing for differences in the probability dimension. The results were consistent with the results in females reported here for the psychometric function (Supplementary Fig. S5), as well as for the tests of economic rationality^53^. Such a high, quantitative predictive value is especially helpful when selecting appropriate reward stimuli for a desired discrimination performance.

The non-linear processing of probability is rarely considered in other animal studies; instead, perfect linear representation is sometimes assumed^60^. However, the non-linearity of the evaluation itself can lead to suboptimal choice^55^ and violations of economic rationality^60^, especially when, as in this study, differences in one dimension influence choice more strongly than differences in another dimension. Consistent with this interpretation, studies of humans and other primates suggest that when stimuli are numerically represented or can be evaluated on a graded continuum in both dimensions (a pre-condition for proportional processing), the evaluation of multi-dimensional options leads to violations of the principles of economic rationality^61^ and references therein). In contrast, when stimuli differ in dimensions such as shape and colour, no consistent violations of economic rationality are reported^61^. It remains to be clarified how mice respond to simultaneous differences in more than one reward dimension and whether these conform to theoretical expectations^60^ under rigorous examination.

The experimental design of the current study allowed the monitoring of exploration behaviour by tracking the sampling rates of mice at the irrelevant dispensers. Although non-rewarding dispensers were visited 20–30% of the time (upper asymptotes in Fig. 5), the optimal strategy would have been to avoid such irrelevant dispensers altogether. However, since foraging animals face the exploration-exploitation dilemma, they must sample options in order to continually gather information about the current state of the environment. This is likely an adaptive behaviour in nature, where, unlike in the laboratory, choice options may change and be unpredictable. The sampling rate was not affected by the difficulty of the discrimination task (Table 4). However, consistent with the matching law^62,63^, mice increased visits to irrelevant dispensers proportionally to the reward there delivered (Table 4), despite the sub-optimality of this behaviour.

We found no consistent evidence for an effect of social learning on exploratory behaviour (sampling rate). Mice in the probability first (*N* = 7), but not in the volume first group (*N* = 8), increased their sampling rates when forced to share their active dispensers with all other mice (Table 5). This can be interpreted as an effect of interference competition caused by crowding at the dispensers. Consequently, mice visited non-rewarding dispensers more frequently. Surprisingly, however, this effect was found only in the smaller (*N* = 7 vs. *N* = 8) of the two groups of mice. Furthermore, after extensive training, only the mice in the larger volume first group, but not in the probability first group, reduced their visitation of the non-rewarding dispensers in the control tests after the main experiment (Table 5). It is possible that group dynamics differed between the two groups, in terms of dominance structure, group cohesion or other uncontrolled (non-)social factors inherent to collective group movement^64,65^; however, these effects must be evaluated in further studies. In any case, although the discrimination performance increased with extensive training in both groups, there was no obvious social effect from forced dispenser-sharing on discrimination performance (Table 5). These results suggest that choices were influenced to a greater extent by individual information than social learning.

In this study, we identified and modelled economic principles that are used by healthy mice to make rational, unidimensional decisions. These results could be used to further understand how decision-making circuits function normally and when disrupted by disease or genetic modification. Subtle deviations from each described model (regularity, transitivity, independence of irrelevant alternatives, psychometric properties) may serve as individual behavioural biomarkers of pathological conditions in mice and humans. In biomedical research, aspects of decision making (e.g., probability discounting) are currently simultaneously scrutinized at behavioural, neurological, molecular and theoretical levels in order to unravel critical endophenotypes leading to complex brain disorders^66–70^.

We have demonstrated the usefulness of using the principles of economic rationality and psychometric analyses for making specific quantitative predictions about choice behaviour in mice. From this basis, which is consistent with normative models of choice, experiments can now be extended to multi-dimensional choice and the use of mouse models of cognitive impairment that may violate principles of economic rationality.

## Electronic supplementary material

Supplementary Figures and Tables
