# Supplementary material for: Principles of Economic Rationality in Mice
Source: Sci Rep. 2017 Dec 12;7:17441. doi: 10.1038/s41598-017-17747-7 (PMC5727109; doi:10.1038/s41598-017-17747-7)
Supplement: Supplementary file 1 — Supplementary Figures and Tables [file 41598_2017_17747_MOESM1_ESM.pdf]

Principles of Economic Rationality in Mice

Marion Rivalan[a], York Winter[a*], Vladislav Nachev[a*]

[a] Humboldt University, Berlin, Germany

[*] Correspondence and present address:

Vladislav Nachev, Dept. of Biology, Humboldt University, Philippstr. 13, 10099 Berlin, Germany
e-mail: vladislav.nachev@charite.de
tel.: +49 (0)30 2093 479 42 fax: +49 (0)30 2093 479 55

Marion Rivalan, Dept. of Biology, Humboldt University, Philippstr. 13, 10099 Berlin, Germany
e-mail: marion.rivalan@charite.de
tel.: +49 (0)30 2093 479 42 fax: +49 (0)30 2093 479 55

York Winter, Dept. of Biology, Humboldt University, Philippstr. 13, 10099 Berlin, Germany
e-mail: york.winter@charite.de
tel.: +49 (0)30 2093 479 40 fax: +49 (0)30 2093 479 55

Supplementary Information

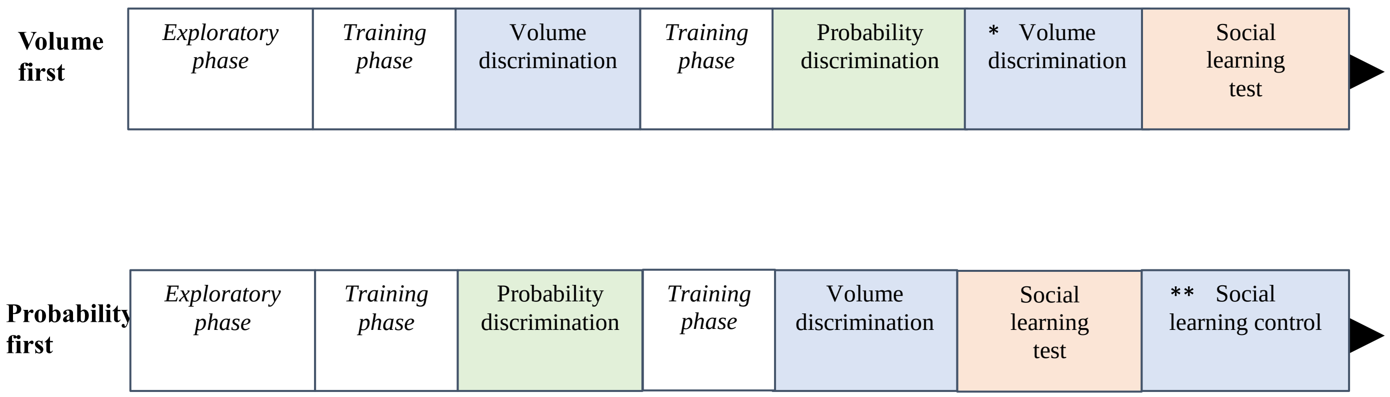

Supplementary Figure S1. Sequence of experimental phases for the mice in the volume first (top row) and probability first groups (bottom row).
Mice remained for different numbers of days in the first two phases, but starting with the first discrimination phase were in synchronous phases.

* Repeats of several volume discrimination conditions, including the AD0 condition, which acted as a control for the social learning test.
** A repeat of the AD0 condition only. There was a single night of *ad libitum* water access before this phase.

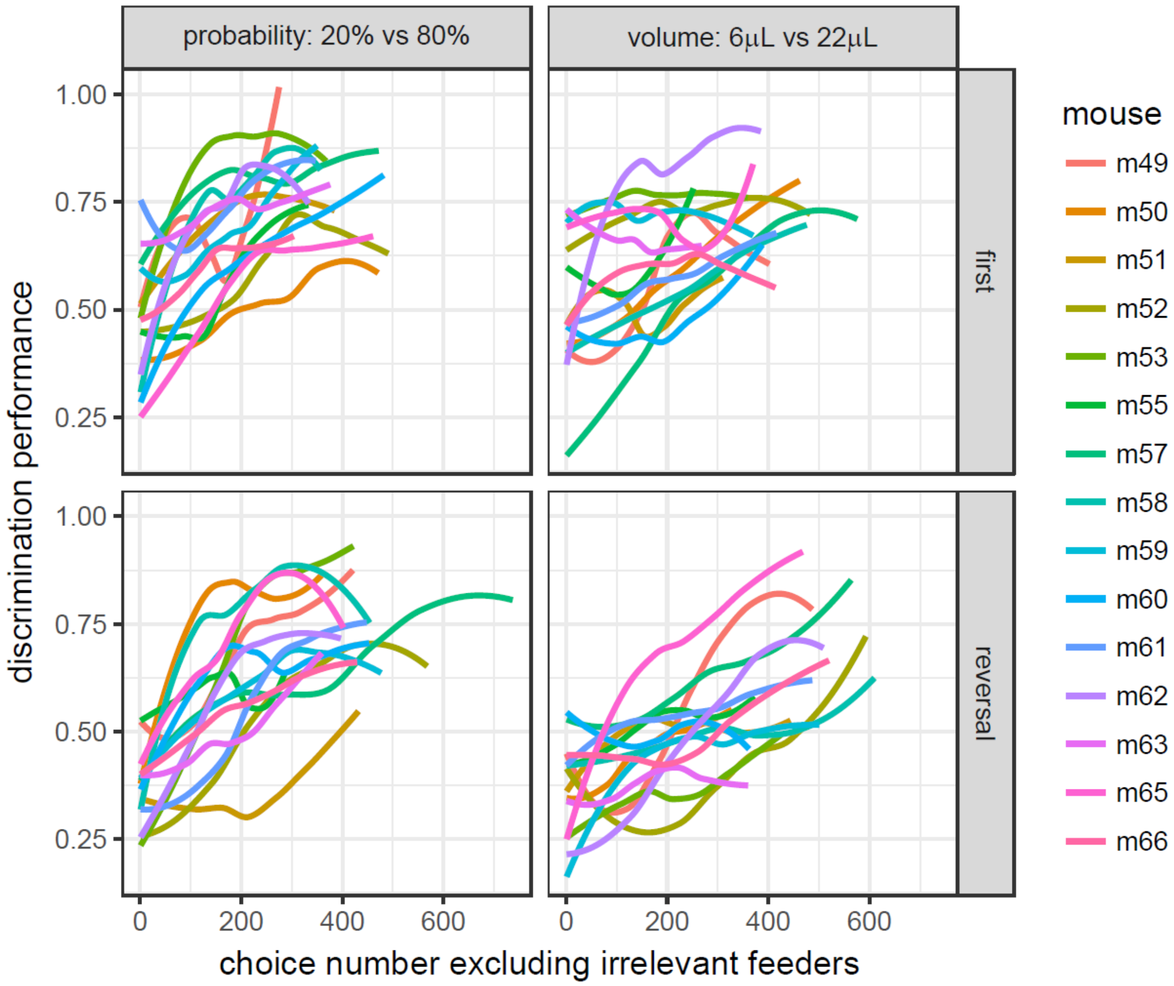

Supplementary Figure S2. Individual learning curves of all mice from the drinking sessions with the highest relative intensity (1.2, condition AD0). Curves show how discrimination performance (visits to dispenser with high profitability over visits at dispensers with high and medium profitabilities) increased with choice number, where only choices to the relevant dispensers were considered. Columns correspond to the reward dimension (probability or volume) and rows, to the first or second (reversal) drinking session of the same experimental condition (AD, see Methods for details). Data were smoothed using LOESS.

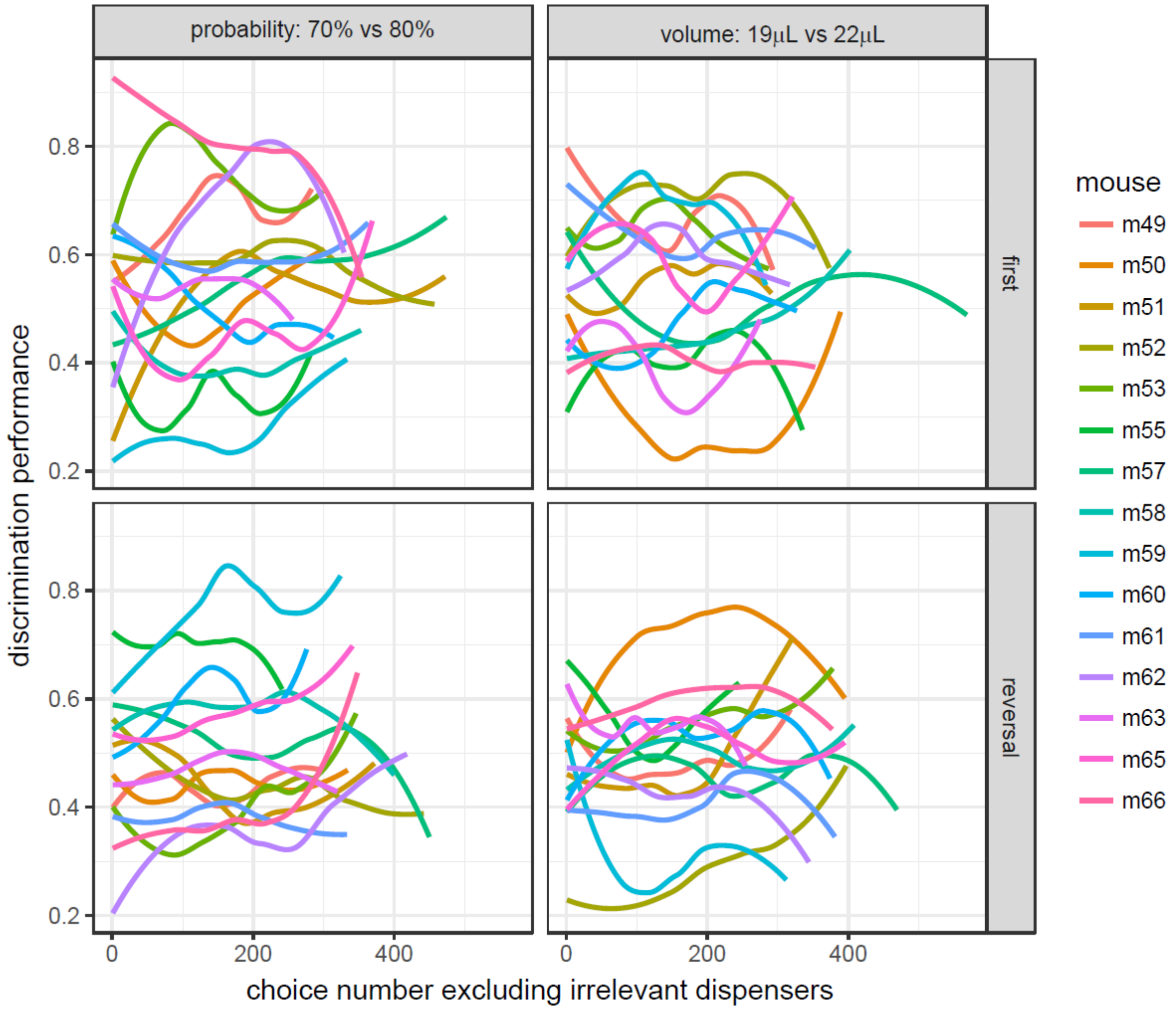

Supplementary Figure S3. Individual learning curves of all mice from the sessions with the smallest relative intensity (0.13, condition AB0). Same notation as in Fig. S2.

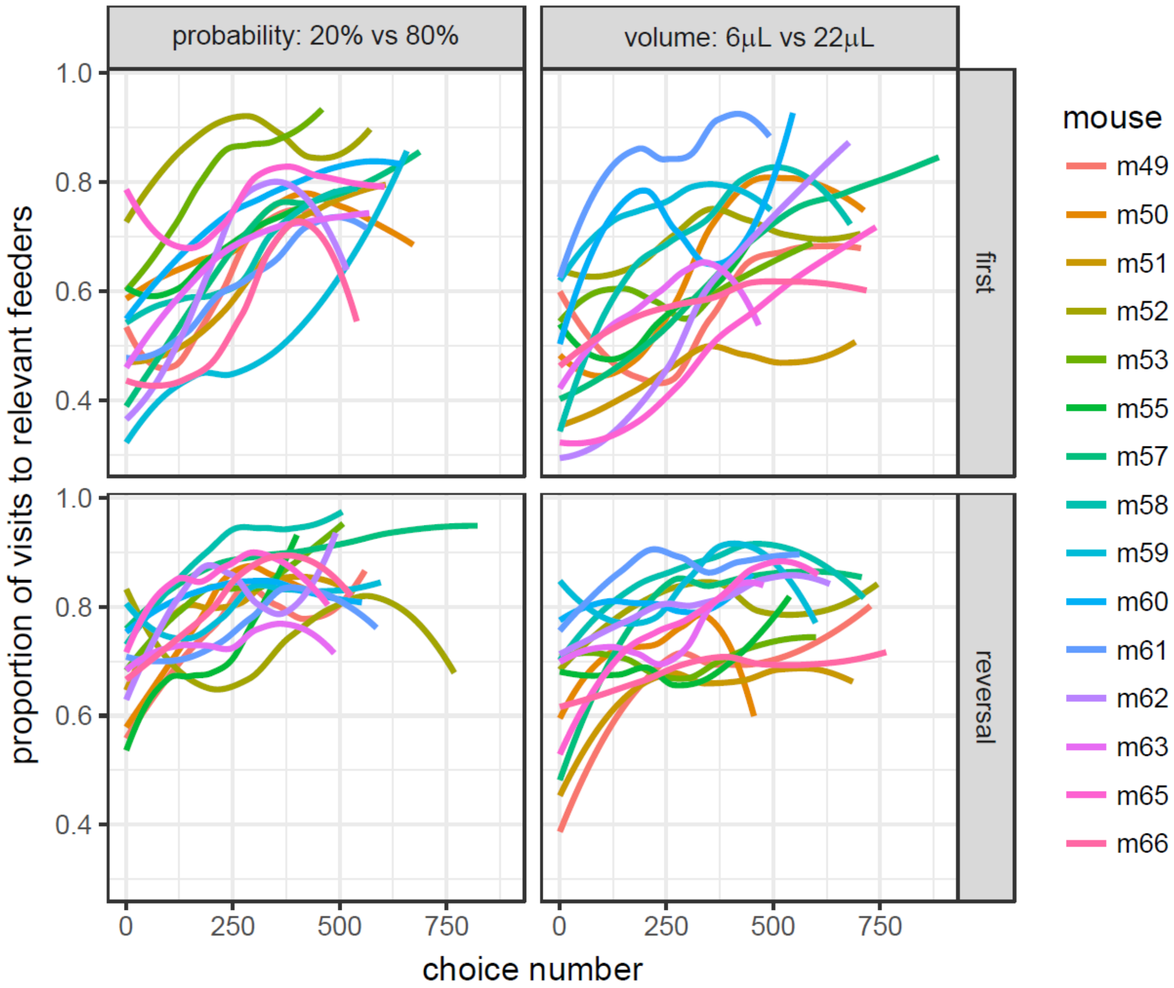

Supplementary Figure S4. Individual learning curves of all mice mice from the drinking sessions with the highest relative intensity (1.2, condition AD0). Curves show how the proportion of visits to the relevant (activated) dispensers increased with choice number. Columns correspond to the reward dimension (probability or volume) and rows, to the first or second (reversal) drinking session of the same experimental condition (AD0, see Methods for details). Data were smoothed using LOESS. Generally, the level achieved on both the first and the reversal day was the same. At the beginning of a drinking session mice explored non-rewarding dispensers more than towards the end.

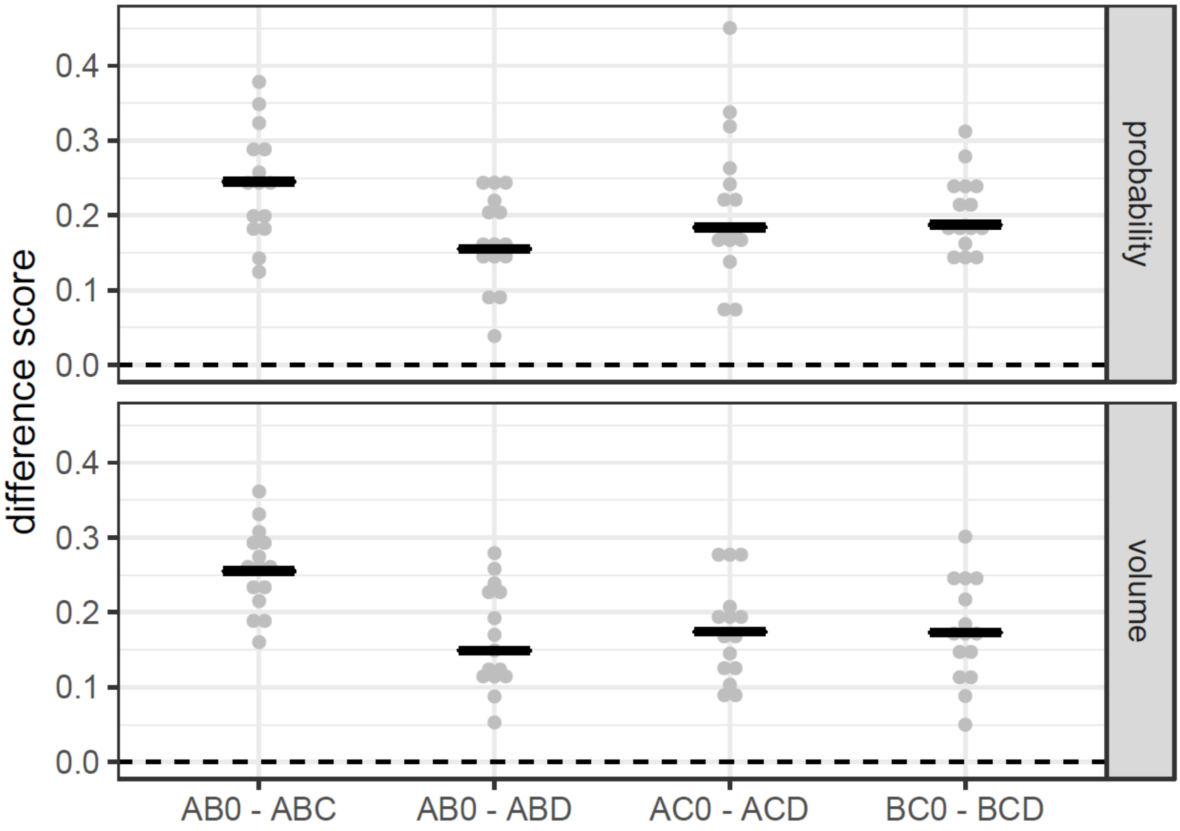

Supplementary Figure S5. Mouse choice complies with the principle of regularity. The plot differs from Fig. 4 in the calculation of the difference score. The difference scores (circles) here are differences between the discrimination performance in binary conditions (AB0, AC0, and BC0) and the visits at the most profitable dispenser as a proportion of all visits, including visits at the irrelevant dispensers, in trinary conditions (ABC, ABD, ACD, and BCD). Since all of the difference scores were positive (above dashed line at 0), no violation of regularity was observed. Thick lines give the medians over all mice ($N = 15$). Rows correspond to the reward dimensions (probability or volume).

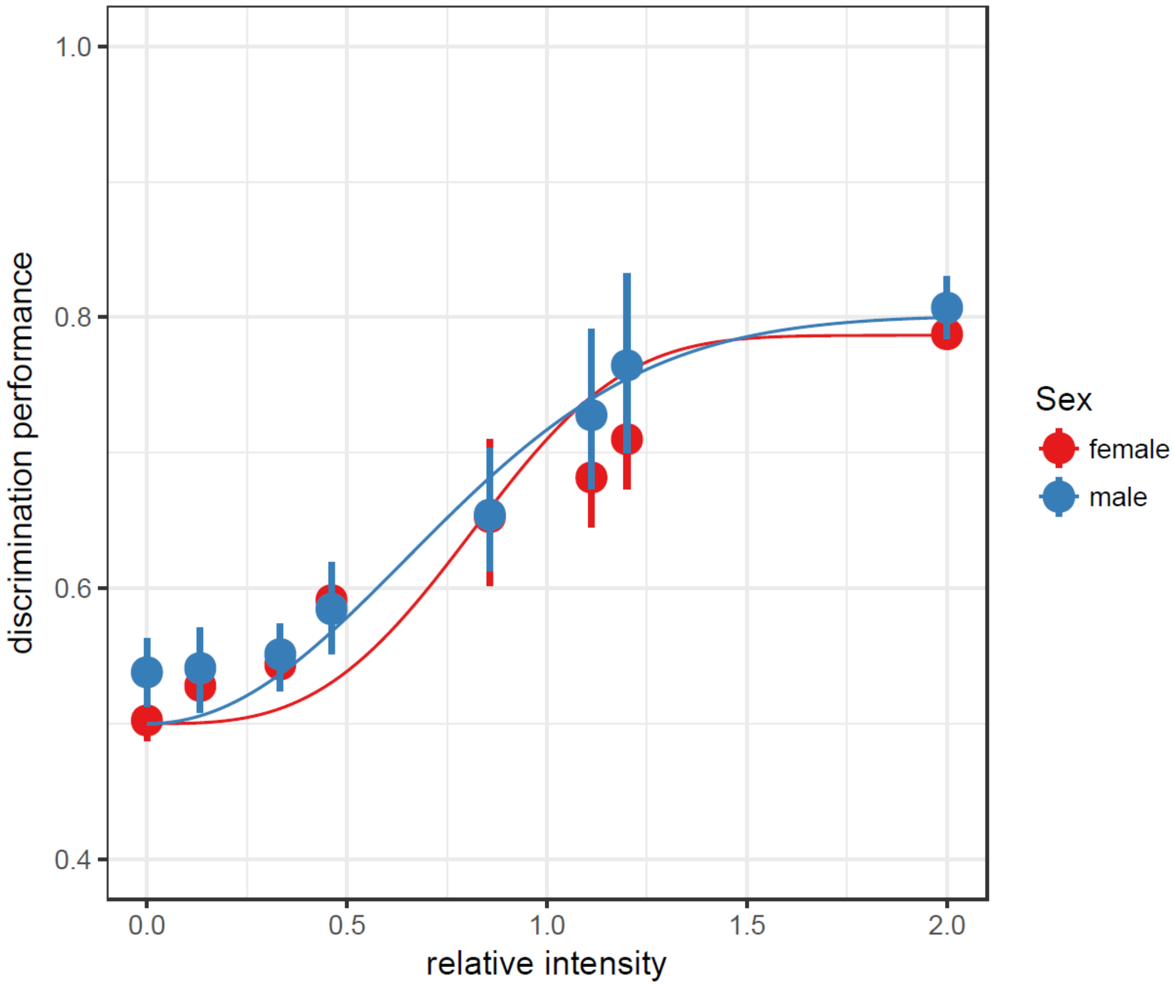

Supplementary Figure S6. Psychometric functions for probability discrimination in male (blue, *N* = 8) and female (red, *N* = 15) mice. Symbols give the average discrimination performances over the groups, and whiskers, the mean standard errors from bootstraps. Lines give the psychometric functions with parameters equal to the average parameters of the individually fitted psychometric functions. Welch's *t* tests revealed no significant differences in lapse rate, threshold, and slope between the two groups.

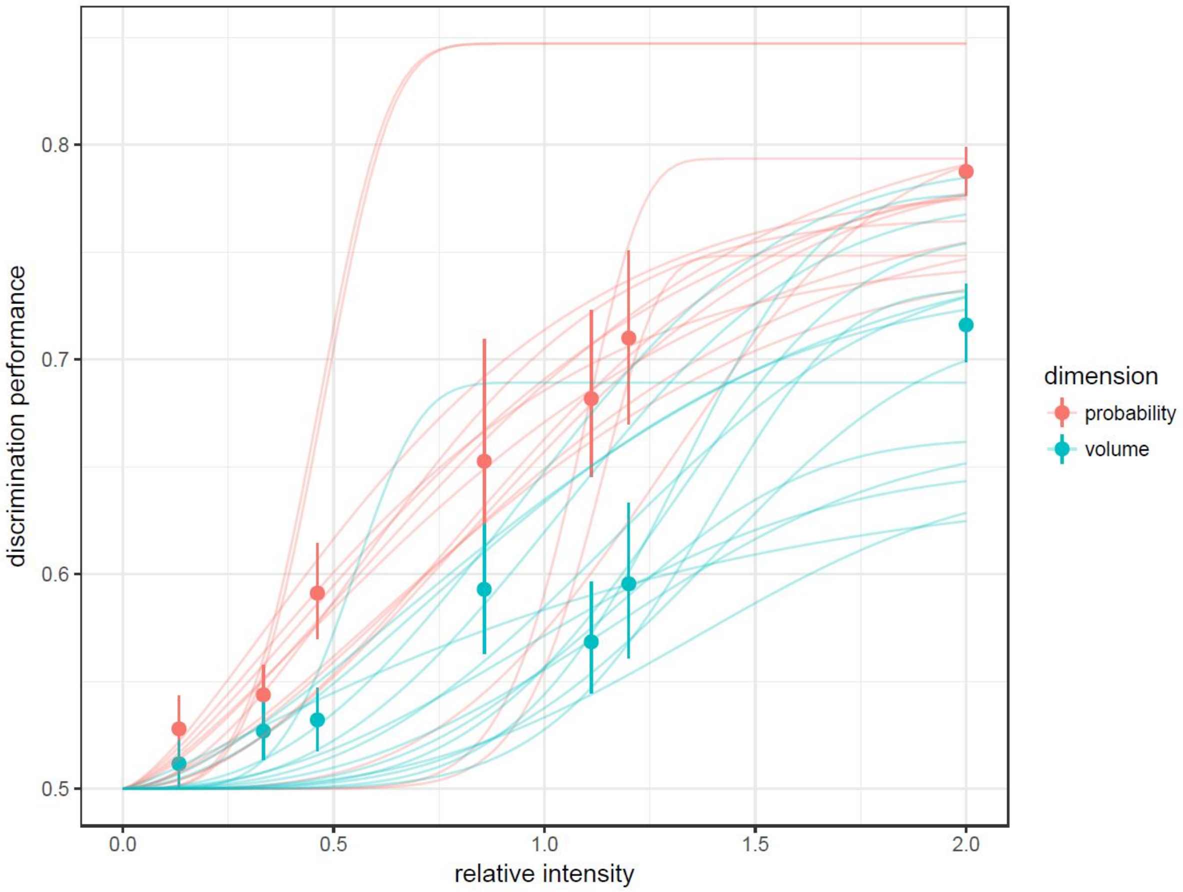

Supplementary Figure S7. Individual psychometric functions for probability (red) and volume discrimination (blue) of female mice (*N* = 15). Symbols give the average discrimination performances over the dimensions, and whiskers, the mean standard errors from bootstraps. Lines give the individually fitted psychometric functions.

Supplementary Table S8. Psychometric function parameters

Reward dimension	Individual	Threshold	Slope	Lapse rate
Probability	m49	0.60	0.82	0.44
Probability	m50	0.61	0.80	0.51
Probability	m51	1.29	0.96	0.40
Probability	m52	0.75	0.66	0.41
Probability	m53	0.46	3.00	0.31
Probability	m55	0.94	0.73	0.42
Probability	m57	0.47	3.00	0.31
Probability	m58	1.07	3.00	0.41
Probability	m59	0.91	0.77	0.43
Probability	m60	1.11	3.00	0.50
Probability	m61	0.86	0.66	0.50
Probability	m62	0.83	0.63	0.37
Probability	m63	0.79	0.62	0.45
Probability	m65	0.67	0.98	0.47
Probability	m66	0.90	0.66	0.47
Probability	Mean ± s.e.	0.82 ± 0.06	1.35 ± 0.27	0.43 ± 0.02
Volume	m49	0.54	3.00	0.62
Volume	m50	1.20	0.79	0.68
Volume	m51	1.03	0.77	0.70
Volume	m52	1.29	1.09	0.48
Volume	m53	1.36	1.41	0.54
Volume	m55	1.39	0.96	0.58
Volume	m57	0.99	0.86	0.42
Volume	m58	0.95	0.59	0.49
Volume	m59	1.19	0.80	0.52
Volume	m60	1.37	0.78	0.71
Volume	m61	1.30	1.41	0.45
Volume	m62	1.07	0.89	0.45

Volume	m63	0.83	0.51	0.71
Volume	m65	0.90	0.71	0.53
Volume	m66	1.15	1.05	0.67
Volume	Mean ± s.e.	1.10 ± 0.06	1.04 ± 0.15	0.57 ± 0.03

s.e. standard error
